# Rare but Relevant: Assessing Variants in Dystonia-linked Genes in Parkinson’s Disease

**DOI:** 10.1101/2025.07.10.25330831

**Published:** 2025-07-11

**Authors:** Lara M. Lange, Zih-Hua Fang, Laurel Screven, Ai Huey Tan, Roy N. Alcalay, Rim Amouri, Roberta Bovenzi, Matilda Fenn, Joshua L. I. Frost, Joseph Jankovic, Simona Jasaityte, Zane Jaunmuktane, Beomseok Jeon, Ignacio Juan Keller Sarmiento, Rejko Krüger, Gregor Kuhlenbäumer, Chin-Hsien Lin, Lukas Pavelka, Maria Teresa Periñan, Samia Ben Sassi, Tommaso Schirinzi, Jung Hwan Shin, Joshua M. Shulman, Yi Wen Tay, Ryan Uitti, Tom Warner, Zbigniew K. Wszolek, Lesley Wu, Ruey-Meei Wu, Kirsten E. Zeuner, Cornelis Blauwendraat, Andrew Singleton, Niccolò E. Mencacci, Huw R. Morris, Shen-Yang Lim, Katja Lohmann, Christine Klein

**Affiliations:** 1 Laboratory of Neurogenetics, National Institute on Aging, Bethesda, Maryland, USA; 2 Institute of Neurogenetics, University of Luebeck, Luebeck, Germany; 3 German Center for Neurodegenerative Diseases (DZNE), Tubingen, Germany; 4 Global Parkinson’s Genetics Program (GP2), Maryland, USA; 5 Division of Neurology, Department of Medicine, Faculty of Medicine, University of Malaya, Kuala Lumpur, Malaysia; 6 Neurological Institute, Tel Aviv Sourasky Medical Center, Tel Aviv, Israel; Department of Neurology, Columbia Irving Medical School, New York, NY, USA; 7 National Institute Mongi Ben Hamida of Neurology, Tunis, Tunisia; 8 Neurology Unit, Department of Systems Medicine, Tor Vergata University of Rome, Rome, Italy; 9 Department of Neurology, Northwestern University Feinberg School of Medicine, Chicago, IL, USA; 10 Department of Clinical and Movement Neurosciences, UCL Queen Square Institute of Neurology, London, UK; 11 Parkinson’s Disease Center and Movement Disorders Clinic, Department of Neurology, Baylor College of Medicine, Houston, TX, USA; 12 Queen Square Brain Bank for Neurological Disorders, UCL Queen Square Institute of Neurology, London, WC1N 1PJ, UK; 13 Division of Neuropathology, National Hospital for Neurology and Neurosurgery, University College London NHS Foundation Trust, London, UK, WC1N 3BG; 14 Department of Neurology, Seoul National University Hospital, Seoul, South Korea; 15 Luxembourg Centre for Systems Biomedicine (LCSB), University of Luxembourg, Luxembourg; 16 Luxembourg Institute of Health (LIH), Strassen, Luxembourg and Centre Hospitalier de Luxembourg (CHL), Luxembourg; 17 University Hospital Schleswig-Holstein (UKSH), Campus Kiel, Kiel, Germany; 18 Department of Neurology, National Taiwan University Hospital, Taipei, Taiwan; 19 Centre for Preventive Neurology, Wolfson Institute of Population Health, Queen Mary University of London, London, United Kingdom.; 20 Unidad de Trastornos del Movimiento, Servicio de Neurología y Neurofisiología Clínica, Instituto de Biomedicina de Sevilla, Hospital Universitario Virgen del Rocío/Consejo Superior de Investigaciones Científicas (CSIC)/Universidad de Sevilla, Seville, Spain.; 21 Duncan Neurological Research Institute, Texas Children’s Hospital, Houston, TX, USA; 22 Mayo Clinic, Jacksonville, FL, USA; 23 Coalition for Aligning Science, Chevy Chase, Maryland, USA; 24 UCL Movement Disorders Centre, University College London, London, UK

**Keywords:** Dystonia, Monogenic, Parkinson’s disease, *GCH1*, *VPS16*

## Abstract

**Background:**

Dystonia and Parkinson’s disease (PD) show clinical and genetic overlap, but the relevance of dystonia gene variants in PD remains unclear.

**Objective:**

To assess the frequency of dystonia-linked pathogenic variants in PD.

**Methods:**

We screened sequencing data from 15,738 individuals (7,851 PD, 4,287 atypical parkinsonism, and 3,600 unaffected) from GP2 and AMP-PD for variants in genes linked to isolated dystonia, dystonia-parkinsonism, and myoclonus-dystonia.

**Results:**

Pathogenic variants were only identified in PD patients. Forty-five PD individuals (0.57%) carried 26 distinct (likely) pathogenic variants in nine dystonia-linked genes, most frequently in *GCH1*, followed by *VPS16*.

**Conclusion:**

Though rare, pathogenic variants in dystonia-linked genes are present in clinically and pathologically diagnosed PD. Our results reinforce *GCH1* as a PD-relevant gene with clinical implications, while variants identified in other genes are rare and of sometimes uncertain relation to the PD phenotype.

## Introduction

Dystonia is a clinically heterogeneous disorder. Its etiology includes nervous system pathologies, acquired causes, and genetic factors. (1) To date, variants in over 400 genes have been linked to different forms of dystonia, though the majority are associated with more complex and broader neurological presentations. (2–7) The term “dystonia” is not only used to refer to a disease entity itself but also to describe a symptom as part of another neurological disorder. For example, dystonic symptoms are frequently reported in individuals with Parkinson’s disease (PD), either related to dopaminergic treatment and motor fluctuations or as an initial disease manifestation, especially in early-onset PD. (8,9) Dystonic symptoms are also commonly encountered in atypical parkinsonism, though the dystonic features typically differ from those in PD. Interestingly, previous screening studies of PD patients identified carriers of pathogenic variants in genes primarily linked to dystonia, most frequently *GCH1*. (10,11)

Moreover, several neurogenetic conditions include features of both dystonia and parkinsonism, either individually or combined, i.e., where dystonia and parkinsonism are equally prominent. Based on this phenotypic and genetic overlap between dystonia and parkinsonism, this study aimed to assess the frequency of pathogenic variants in dystonia genes in individuals with PD and atypical parkinsonism by leveraging large-scale sequencing data from the Global Parkinson’s Genetics Program (GP2; https://gp2.org/) (12,13) and the Accelerating Medicines Partnership - Parkinson’s disease (AMP-PD; https://www.amp-pd.org/).

## Methods

**Figure 1.**
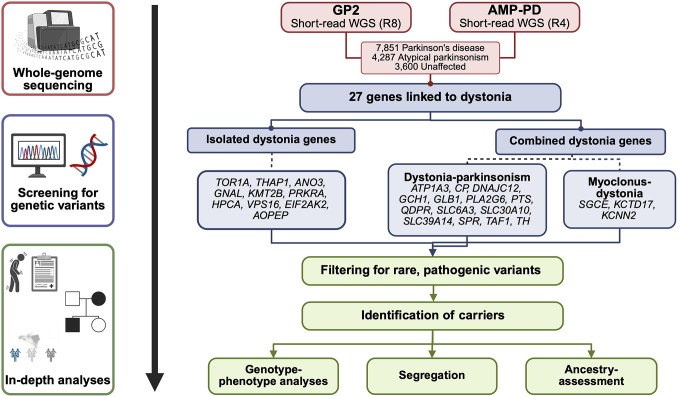
Study design and workflow. We screened short-read whole-genome sequencing (WGS) data from the Global Parkinson’s Genetics Program (GP2; Release 8 [R8]) and the Accelerating Medicines Partnership - Parkinson’s disease (AMP-PD, Release 4 [R4]) for known variants in genes linked to different forms of dystonia. We included genes linked to isolated as well as combined dystonia phenotypes, the latter including dystonia-parkinsonism and myoclonus-dystonia. Only rare variants predicted to be pathogenic or likely pathogenic were included in further analyses. For identified carriers, we evaluated genotype-phenotype correlations, investigated segregation, and assessed ancestry distributions, where possible. This figure was created with BioRender.

Figure 1 illustrates our workflow. We analyzed short-read sequencing data from 15,738 individuals of eleven genetically determined ancestries from GP2’s Data Release 8 (DOI 10.5281/zenodo.13755496) and AMP-PD’s Release 4, including 7,851 PD patients, 4,287 individuals with atypical parkinsonism, and 3,600 unaffected individuals (see Supplementary Material).

We investigated variants in genes linked to dystonia following the recommendations of the *MDS Task Force on the Nomenclature of Genetic Movement Disorders* (2): i) isolated dystonia: *ANO3, AOPEP, EIF2AK2, GNAL, HPCA, KMT2B, PRKRA, THAP1, TOR1A*, and *VPS16*, ii) dystonia-parkinsonism: *ATP1A3, CP, DNAJC12, GCH1, GLB1, PLA2G6, PTS, QDPR, SLC6A3, SLC30A10, SLC39A14, SPR, TAF1*, and *TH*, and iii) myoclonus-dystonia: *SGCE, KCTD17*, and *KCNN2*. We filtered for rare (gnomAD minor allele frequency ≤1%) variants classified as pathogenic/likely pathogenic according to ClinVar (https://www.ncbi.nlm.nih.gov/clinvar/) and ACMG criteria (14) (see Supplementary Material).

## Data and Code Availability

Data used in the preparation of this article were obtained from the Global Parkinson’s Genetics Program (GP2; https://gp2.org). Specifically, we used Tier 2 data from GP2 release 8 (DOI: 10.5281/zenodo.13755496). Tier 1 data can be accessed by completing a form on the Accelerating Medicines Partnership in Parkinson’s Disease (AMP-PD) website (https://amp-pd.org/register-for-amp-pd). Tier 2 data access requires approval and a Data Use Agreement signed by your institution. AMP-PD data can be accessed through the AMP-PD website (https://amp-pd.org). Qualified researchers are encouraged to apply for direct access to the data through AMP-PD.

All code generated for this article, and the identifiers for all software programs and packages used, are available on GitHub (https://github.com/GP2code/dystonia-genes-inPD) and were given a persistent identifier via Zenodo (DOI 10.5281/zenodo.15676002).

## Results

We identified 45 individuals from six ancestries carrying 26 distinct pathogenic/likely pathogenic variants in nine dystonia-linked genes. The identified pathogenic/likely pathogenic variants and their pathogenicity evaluation are summarized in Table 1. All 45 individuals were diagnosed with PD (n=45/7,851; 0.57%), while no carriers were diagnosed with atypical parkinsonism (n=0/4,287; 0%) or among unaffected individuals (n=0/3,600; 0%). The majority (n=43) were carriers of heterozygous variants in genes linked to dominantly inherited dystonia genes (*ATP1A3, GCH1, SGCE, KMT2B, THAP1, TOR1A*, and *VPS16*), while only one carrier of a homozygous *PLA2G6* variant and one of compound-heterozygous *PTS* variants (Supplementary Figure 3) were identified.

Overall, variants in *GCH1* were the most frequent (n=21/45, 46.7%), followed by *VPS16* (n=12/45, 26.7%). Notably, there was one ancestry-specific recurrent variant in both these genes: *GCH1* p.Lys224Arg, carried by ten European-ancestry individuals, including two siblings, and *VPS16* p.Asn52Lys, carried by eight East Asian individuals. According to gnomAD, both variants are more frequent in these respective ancestral groups compared to other ancestries; however, the frequency in PD cases in our study was higher compared to ancestry-matched controls (Supplementary Table 2). With respect to additional variants in PD-linked genes, seven of the 45 individuals carried *GBA1* or *LRRK2* risk variants, and two carried heterozygous pathogenic variants in recessively inherited PD genes (Table 2), but none harbored a disease-explaining variant in an established PD-linked gene (including *LRRK2, SNCA, VPS35, RAB32, PINK1, PRKN*, and *PARK7/DJ-1*).

Table 2 displays the demographic and clinical characteristics of identified variant carriers. The majority with available data on treatment showed a good response to levodopa (n=25/26, missing for n=19). The median age at PD motor symptom onset or PD diagnosis across all carriers was 47 years (IQR: 38–59 years), ranging from 27 to 83 years. Fourteen carriers showed variable signs of dystonia (information unavailable for n=19); which was believed to be related to motor fluctuations due to dopaminergic medications (OFF dystonia) in a subset. While 17 (42.5%, unknown for n=5) carriers had a positive family history of PD, only two individuals had family members with dystonia (unknown for n=19).

Interestingly, one late-onset PD patient (age at onset >70 years) without dystonia, carrying the pathogenic *TOR1A* p.Glu303del variant, had a mother with PD, but also one daughter and two grandchildren with dystonia (Supplementary Figure 2A). Unfortunately, no additional family members were available for genetic testing.

We also identified a pathogenic *THAP1* variant (p.Lys73Thr) in an individual and her mother (Supplementary Figure 2B), both diagnosed with PD and slight foot OFF-dystonia. Interestingly, the mother’s first motor symptom (age at onset >65 years) were foot cramps, while her daughter showed no additional signs of possible dystonia, and had a younger age at onset (<38 years of age).

In addition to these 45 individuals, we identified ten carriers of single heterozygous pathogenic variants in recessively inherited dystonia genes, including *AOPEP* (n=5), *SPR* (n=4), and *HPCA* (n=1) (Supplementary Table 1). We did not detect any pathogenic/likely pathogenic variants in *ANO3, EIF2AK2, GNAL, PRKRA*, *CP, DNAJC12, GLB1, QDPR, SLC6A3, SLC30A10, SLC39A14, TAF1, TH*, *KCTD17*, and *KCNN2*. However, 613 individuals carried rare variants of uncertain significance across all investigated genes (580 with PD, eight with atypical parkinsonism, and 25 unaffected).

## Discussion

Pathogenic variants in dystonia-linked genes were found in less than 1% of PD patients. Despite being rare, they were enriched in PD patients compared to controls and individuals with atypical parkinsonism, where no such variants were identified.

Not surprisingly and in line with previous studies (10,11), variants in *GCH1* were relatively frequent. *GCH1* is known to cause dystonia and parkinsonism, independently or combined. According to a systematic review, 86% of heterozygous *GCH1* carriers present with dystonia to a variable extent, either isolated or combined dystonia-parkinsonism, while 11% have only parkinsonism. (15) Several case series reported PD patients harboring pathogenic *GCH1* with abnormal DaTscan imaging, indicating nigrostriatal dopaminergic deficits consistent with neurodegenerative parkinsonism (11,16,17). Another study suggested *GCH1* variants may lead to parkinsonism by unmasking subclinical nigral pathology, not by causing nigral neurodegeneration. (18) Additionally, according to the most recent PD genome-wide association studies, common variants were nominated as the likely risk factor in the *GCH1* locus. (19,20) Notably, we identified one recurrent *GCH1* variant (p.Lys224Arg) only present in European-ancestry individuals. Although this variant has a conflicting ClinVar interpretation, it was only found in PD patients and absent from controls in our study, and it was significantly more frequent in European-ancestry PD cases from our study compared to ancestry-matched controls obtained from gnomad (Supplementary Table 2). Further, it was present in two PD patients from the same family (sisters), supporting its pathogenic role. In addition to *GCH1*, variants in genes linked to dystonia-parkinsonism (i.e., *ATP1A3, PLA2G6* and *PTS*) collectively accounted for more than half of the identified carriers overall (24/45, 53%).

Interestingly, 47% (n=21/45) of all identified carriers harboured variants in isolated dystonia genes, where parkinsonism is unexpected. (21,22) Amongst those genes was *VPS16*, with the second largest number of pathogenic variants (n=9) observed in this study. We identified one recurrent *VPS16* variant, exclusively present in East Asian individuals. However, although predicted to be pathogenic on ClinVar and reported as disease-causing in Chinese dystonia patients in the homozygous state (23), the overall pathogenicity evaluation remains partially conflicting since this variant is relatively frequent in East Asians controls. In our study, the frequency in East Asian PD cases was higher compared to ancestry-matched controls obtained from gnomad, though this trend was not statistically significant (Supplementary Table 2). Overall, whether these findings in isolated dystonia genes indicate a causal relationship and contribute to the parkinsonian phenotype (variable clinical expressivity), remains elusive. Another, probably more likely, explanation might be that these are incidental findings reflecting reduced penetrance (for dystonia), a phenomenon well known in several monogenic forms of dystonia, especially in DYT-*THAP1* (penetrance ~50%) and DYT-*TOR1A* (penetrance ~30%).

Finally, we identified one carrier of a pathogenic variant in the myoclonus-dystonia gene *SGCE*. Notably, due to maternal imprinting, *SGCE* variants show significantly reduced penetrance when inherited maternally, and the phenotype only develops if the variant is inherited paternally. Unfortunately, no additional family members were available for genetic testing, and we were unable to further investigate the inheritance pattern.

This study has some limitations. The availability of clinical data for samples obtained from AMP-PD was very limited; detailed data on dystonia were not available, thereby not allowing us to perform meaningful genotype-phenotype analyses. Further, family members of identified variant carriers were unavailable for genetic testing within this study; precluding us from performing segregation analyses, which would be helpful in assessing pathogenicity, especially in case of genes with reduced penetrance.

In conclusion, pathogenic variants in dystonia-linked genes were present, albeit rare, among PD cases and absent in individuals with atypical parkinsonism and controls. While misdiagnosis cannot be excluded, available clinical and pathological data support a neurodegenerative disease in most cases. The enrichment in PD patients compared to controls suggest that dystonia gene variants may predispose to PD and that there may be potential biological overlap and shared pathways between dystonia and PD. Importantly, our results, alongside previous evidence from screening studies and GWAS, reinforce *GCH1* as a PD-relevant gene holding clinical implications. Similarly, other genes associated with dystonia-parkinsonism may have clinical relevance for PD patients, whereas variants in isolated or myoclonus-dystonia genes more likely represent incidental findings, potentially due to reduced penetrance. Collectively, our results highlight the need for careful interpretation and counseling in clinical genetics regarding a possible role of pathogenic dystonia gene variants in PD patients.

## Supplementary Material

Supplement 1

## Figures and Tables

**Table 1.** Overview of identified pathogenic and likely pathogenic variants in dystonia-linked genes.

Gene	Chromosomal position	cDNA change	Protein change	Variant type	Zygosity	Pathogenicity	Franklin	Varsome	MDSGene	ClinVar	CADD	GnomAD AF	n carrier
**Pathogenic variants in combined dystonia-parkinsonism genes**													
***ATP1A3***	chr19:41978053:T:C	c.1826A>G	p.Asp609Gly	missense	het	P/LP	Likely pathogenic	Likely pathogenic	NA	NA	32	NA	1
***GCH1***	chr14:54844023:C:G	c.747G>C	p.Arg249Ser	missense	het	P/LP	Likely pathogenic	VUS	Probably pathogenic	Uncertain significance	22.7	NA	1
	chr14:54844067:G:A	c.703C>T	p.Arg235Trp	missense	het	P/LP	Likely pathogenic	VUS	Probably pathogenic	NA	32	NA	1
	chr14:54844080:C:T	c.690G>A	p.Met230Ile	missense	het	P/LP	Likely pathogenic	Likely pathogenic	Possibly pathogenic	NA	26.2	NA	1
	chr14:54844099:T:C	c.671A>G	p.Lys224Arg	missense	het	P/LP	Pathogenic	Likely benign	Possibly pathogenic	Conflicting	21.3	0,000393846	10
	chr14:54844141:T:A	c.629A>T	p.His210Leu	missense	het	P/LP	Likely pathogenic	Likely pathogenic	NA	NA	27.5	NA	1
	chr14:54845766:AC:A	c.626+1del	NA	splicing	het	P/LP	Likely pathogenic	Likely pathogenic	NA	NA	32	NA	1
	chr14:54845784:C:T	c.610G>A	p.Val204Ile	missense	het	P/LP	Pathogenic	VUS	Possibly pathogenic	Conflicting	24.9	0,000124752	3
	chr14:54845787:C:T	c.607G>A	p.Gly203Arg	missense	het	P/LP	Pathogenic	Pathogenic	Probably pathogenic	Pathogenic	29.1	NA	1
	chr14:54845837:G:C	c.557C>G	p.Thr186Arg	missense	het	P/LP	Likely pathogenic	Pathogenic	NA	NA	28.8	NA	1
	chr14:54902414:C:A	c.250G>T	p.Glu84*	nonsense	het	P/LP	Pathogenic	Pathogenic	Probably pathogenic	Uncertain significance	41	NA	1
***PLA2G6***	chr22:38132917:C:A	c.991G>T	p.Asp331Tyr	missense	hom	P/LP	Pathogenic	VUS	NA	Pathogenic	26.0	0,0000262509	1
***PTS***	chr11:112233178:C:T	c.259C>T	p.Pro87Ser	missense	comphet	P/LP	Pathogenic	Likely pathogenic	Probably pathogenic	Pathogenic	23.3	0,000013138	1
	chr11:112233191:A:G	c.272A>G	p.Lys91Arg	missense	comphet	P/LP	Pathogenic	Likely pathogenic	Probably pathogenic	Pathogenic	22.8	NA	1
**Pathogenic variants in myoclonus-dystonia genes**													
***SGCE***	chr7:94603289:C:G	c.825+1G>C	NA	splicing	het	P/LP	Likely pathogenic	Likely pathogenic	Probably pathogenic	NA	33	NA	1
**Pathogenic variants in isolated dystonia genes**													
***KMT2B***	chr19:35728121:G:A	c.4521G>A	p.Trp1507*	nonsense	het	P/LP	Likely pathogenic	Likely pathogenic	NA	NA	43	NA	1
***THAP1***	chr8:42839235:T:G	c.218A>C	p.Lys73Thr	missense	het	P/LP	Likely pathogenic	VUS	Possibly pathogenic	NA	25.2	0,00000656858	2
	chr8:42843044:G:C	c.51C>G	p.Asp17Glu	missense	het	P/LP	Likely pathogenic	VUS	Possibly pathogenic	NA	13.16	NA	1
	chr8:42843084:G:T	c.11C>A	p.Ser4Tyr	missense	het	P/LP	Likely pathogenic	Likely pathogenic	Possibly pathogenic	NA	25.7	NA	1
***TOR1A***	chr9:129814061:TCTC:T	c.907_909del	p.Glu303del	inframe deletion	het	P/LP	Pathogenic	VUS	NA	Pathogenic/Likely pathogenic	19.47	0,0000525348	2
	chr9:129814108:C:T	c.863G>A	p.Arg288Gln	missense	het	P/LP	Likely pathogenic	VUS	Probably pathogenic	Pathogenic/Likely pathogenic	23.0	0,000059149	1
***VPS16***	chr20:2860067:C:A	c.156C>A	p.Asn52Lys	missense	het	P/LP	VUS	VUS	Probably pathogenic	Pathogenic	18.80	0,00007435	8
	chr20:2860458:C:T	c.379C>T	p.Gln127*	nonsense	het	P/LP	Likely pathogenic	Likely pathogenic	NA	NA	37	NA	1
	chr20:2863064:G:T	c.1332–1G>T	NA	splicing	het	P/LP	Likely pathogenic	Likely pathogenic	NA	Uncertain significance	31	0,0000262902	1
	chr20:2864993:G:GT	c.1943dup	p.Ala649Serfs*16	frameshift	het	P/LP	Likely pathogenic	Likely pathogenic	NA	NA	33	NA	1
	chr20:2866316:G:A	c.2375+1G>A	NA	splicing	het	P/LP	Likely pathogenic	Likely pathogenic	NA	NA	34	NA	1

AF = allele frequency, comphet = compound heterozygous, het = heterozygous, hom = homozygous, NA = not available/not applicable, P/LP = pathogenic/likely pathogenic, VUS = variant of uncertain significance

CADD: Combined Annotation Dependent Depletion; https://cadd.gs.washington.edu/; ClinVar: https://www.ncbi.nlm.nih.gov/clinvar/; GnomAD: https://gnomad.broadinstitute.org/ (version 4.1.0); Franklin: https://franklin.genoox.com; MDSGene: https://www.mdsgene.org/; Varsome: https://varsome.com/

**Table 2.** Demographic and clinical characteristics of identified variant carriers.

ID	Gender	Genetic ancestry	Gene	Variant	Additional genetic finding	Diagnosis	AAO/AAD [1]	Symptom at onset	Levodopa responsive	FH PD	FH Dystonia	Details FH	Signs of Dystonia	Atypical signs
**Pathogenic variants in combined dystonia-parkinsonism genes**														
GP2-ID-1	Male	EUR	*ATP1A3*	p.Asp609Gly		PD	26–30	NA	Yes	No	No	NA	Severe OFF dystonia	NA
GP2-ID-2	Male	EAS	*GCH1*	p.Arg249Ser		PD	66–70	Tremor and slowness	Yes	Yes	No	Brother with PD	No	No; dementia after 10 years disease duration
GP2-ID-3	Female	AAC	*GCH1*	p.Arg235Trp		PD	31–35	Gait disorder	Yes	No	No	NA	Cervical dystonia	Mental retardation
GP2-ID-4	Female	CAS	*GCH1*	p.Met230Ile		PD	41–45	NA	NA	No	NA	NA	NA	NA
GP2-ID-5*	Female	EUR	*GCH1*	p.Lys224Arg		PD	71–75	NA	NA	Yes	NA	Sister with PD	NA	No
GP2-ID-6*	Female	EUR	*GCH1*	p.Lys224Arg		PD	56–60	NA	NA	Yes	NA	Sister with PD	NA	No
GP2-ID-7	Male	EUR	*GCH1*	p.Lys224Arg		PD	36–40	NA	NA	Yes	NA	Father with possible PD (not diagnosed)	NA	NA
AMPP D-ID-1	Female	EUR	*GCH1*	p.Lys224Arg		PD	NA	NA	NA	NA	NA	NA	NA	NA
AMPP D-ID-2	Female	EUR	*GCH1*	p.Lys224Arg		PD	61–65	NA	NA	Yes	NA	NA	NA	NA
GP2-ID-8	Male	EUR	*GCH1*	p.Lys224Arg		PD	66–70	Rigidity and bradykinesia	Yes	Yes	No	Father with PD	No	No
GP2-ID-9	Male	EUR	*GCH1*	p.Lys224Arg	*GBA1* p.Arg296Gln	PD	31–35	Aching Shoulder, dragging left leg	Yes	Yes	No	Paternal uncle with PD	Slight OFF dystonia	No
AMPP D-ID-3	Female	EUR	*GCH1*	p.Lys224Arg		PD	NA	NA	NA	No	NA	NA	NA	NA
AMPP D-ID-4	Male	EUR	*GCH1*	p.Lys224Arg		PD	66–70	NA	NA	No	NA	NA	NA	NA
AMPP D-ID-5	Male	EUR	*GCH1*	p.Lys224Arg		PD	NA	NA	NA	NA	NA	NA	NA	NA
GP2-ID-10	Female	MDE	*GCH1*	p.His210Leu		PD	56–60	Tremor	Yes	Yes	No	Brother with PD	No	No
AMPP D-ID-6	Male	EUR	*GCH1*	c.626+1del		PD	NA	NA	NA	NA	NA	NA	NA	NA
GP2-ID-11	Male		*GCH1*	p.Val204Ile		PD	51–55	Involuntary jaw and tongue movement, rigidity, dragging right foot	Yes	Yes	No	Father with PD	No	No
GP2-ID-12	Male	EUR	*GCH1*	p.Val204Ile		PD	41–45	Loss of dexterity	Yes	No	No	Not applicable	No	No
GP2-ID-13	Male	EUR	*GCH1*	p.Val204Ile		PD	36–40	Action tremor	Yes	No	No	NA	No	No
AMPP D-ID-7	Male	EUR	*GCH1*	p.Gly203Arg		PD	56–60	NA	NA	No	NA	NA	NA	NA
GP2-ID-14	Female	EUR	*GCH1*	p.Thr186Arg		PD	31–35	NA	NA	No	NA	NA	NA	NA
AMPP D-ID-8	Male	EUR	*GCH1*	p.Glu84*		PD	NA	NA	NA	NA	NA	NA	NA	NA
GP2-ID-15	Female	EAS	*PLA2G6*	p.Asp331Tyr	*LRRK2* p.Gly2385Arg	PD	26–30	Right leg slowness	Yes	No	Yes	Consanguineous family	Feet and hand dystonia	Severe depression
GP2-ID-16	Male	EAS	*PTS*	p.Pro87Ser; p.Lys91Arg		PD	41–45	Right leg dystonia	Yes	NA	NA	NA	Right leg dystonia while walking	No
**Pathogenic variants in combined myoclonus-dystonia genes**														
GP2-ID-19	Female	EUR	*SGCE*	c.825+1G>C		PD	36–40	NA	NA	No	NA	NA	NA	NA
**Pathogenic variants in isolated dystonia genes**														
AMPP D-ID-14	Female	AJ	*KMT2B*	p.Trp1507*		PD	56–60	NA	NA	NA	NA	NA	NA	NA
GP2-ID-20*	Female	EUR	*THAP1*	p.Lys73Thr		PD	36–40	Tremor, rigidity	Yes	Yes	No	Mother with PD	Slight OFF dystonia	No
GP2-ID-21*	Female	EUR	*THAP1*	p.Lys73Thr	*PRKN* p.Pro132Thrfs*9 (het)	PD	66–70	Foot Cramps	Yes	Yes	No	Daughter with PD	Slight OFF dystonia	No
GP2-ID-22	Male	EUR	*THAP1*	p.Asp17Glu		PD	36–40	NA	NA	Yes	NA	Mother with parkinsonism	NA	No
GP2-ID-23	Female	EAS	*THAP1*	p.Ser4Tyr		PD	36–40	Gait disturbance	Yes	Yes	No	Brother with PD	No	No
AMPP D-ID-15	Female	EUR	*TOR1A*	p.Glu303del		PD	51–55	NA	NA	No	NA	NA	NA	NA
GP2-ID-24	Male	AJ	*TOR1A*	p.Glu303del		PD	71–75	Spatial difficulties	Yes	Yes	Yes	Mother with PD; Daughter and two granddaughters with dystonia	No	Memory loss, visual hallucination, RBD
GP2-ID-25	Female	EUR	*TOR1A*	p.Arg288Gln	*GBA1* p.Asn409Ser	PD	41–45	Tremor, stiffness in the neck	Yes	Possibly	No	Maternal grandfather with possible PD; Mother with AD	Mild dystonic posture in left arm	no
GP2-ID-26	Male	EAS	*VPS16*	p.Asn52Lys		PD	46–50	Upper limb tremors, bradykinesia	Yes	No	No	Not applicable	Right striatal toe, mild camptocormia and pisa syndrome	No
GP2-ID-27	Male	EAS	*VPS16*	p.Asn52Lys		PD	31–35	Tremor, stiffness, slow movements	Yes#	No	No	Not applicable	Blepharospasm	No
GP2-ID-28	Male	EAS	*VPS16*	p.Asn52Lys	*GBA1* p.Leu483Arg, *PINK1* p.Leu347Pro (het)	PD	46–50	Chest muscle pain, limb tremor	Yes#	No	No	Not applicable	Flexed posture of the left hand, intermittent slight OFF dystonia	No
GP2-ID-29	Female	EAS	*VPS16*	p.Asn52Lys		PD	46–50	Tremor	Yes	No	No	Not applicable	No	No
GP2-ID-30	Female	EAS	*VPS16*	p.Asn52Lys	*GBA1* p.Arg502Cys	PD	46–50	Right hand tremor	No	Yes	No	Father with PD	No	No
GP2-ID-31	Male	EAS	*VPS16*	p.Asn52Lys		PD	56–60	Unk	Yes#	Yes	No	Mother and brother with PD	No	No
GP2-ID-32	Female	EAS	*VPS16*	p.Asn52Lys	*LRRK2* p.Arg1628Pro	PD	31–35	Body weakness associated with cramps	Yes	No	No	Not applicable	Cervical dystonia	Postural hypotension, frequent falls
GP2-ID-33	Male	EAS	*VPS16*	p.Asn52Lys		PD	41–45	Rest tremor, micrographia, slowness in initiating movement	Yes	No	No	Not applicable	No	No
GP2-ID-34	Male	EUR	*VPS16*	p.Gln127*		PD	66–70	NA	NA	Not Reported	NA	NA	NA	Pathology diagnoses: LBD, CAA, AD
GP2-ID-35	Female	EUR	*VPS16*	c.1332–1G>T	*GBA1* RecNciI	PD	36–40	Slowness	Yes	No	No	NA	Limb dystonia (with ON exaggeration)	No
GP2-ID-37	Female	EAS	*VPS16*	p.Ala649Serfs*16		PD	56–60	Tremor	Yes#	Yes	No	Paternal aunt with PD; Multiple family members (e.g., father and brother) with tremor	Leg dystonia (probably in OFF-medication state)	No
GP2-ID-38	Male	EUR	*VPS16*	c.2375+1G>A		PD	81–85	NA	NA	Not Reported	NA	NA	NA	Pathology diagnoses: LBD, ARTAG, FTLD, SVD

AAC = African admixed ancestry, AD = Alzheimer’s disease, AJ = Ashkenazi Jewish, ARTAG = Aging-Related Tau Astrogliopathy, CAS = Central Asian ancestry, CAA = Cerebral Amyloid Angiopathy, EAS = East Asian ancestry, EUR = European, FTLD = Frontotemporal Lobar Degeneration, het = heterozygous, LBD = Lewy Body Dementia, MDE = Middle Eastern ancestry, PD = Parkinson’s disease, RBD = REM sleep behavior disorder, SVD = Small Vessel Disease

[1] When both were available, the AAO is displayed. For those individuals without an available AAO, we indicate the AAD. According to MedRxiv’s regulations, AAO/AAD is provided in ranges.

* The asterisk highlights related individuals within the investigated cohort. GP2-ID-5 and GP2-ID-6 are siblings, and GP2-ID-20 is the daughter of GP2-ID-21.

# This symbol highlights individuals who underwent deep brain stimulation (DBS).
